# Subcutaneous tissue reaction to castor oil bean and calcium hydroxide in rats

**DOI:** 10.1590/S1678-77572010000300014

**Published:** 2010

**Authors:** Samira Esteves Afonso CAMARGO, Sigmar de Mello RODE, Renata Falchete do PRADO, Yasmin Rodarte CARVALHO, Carlos Henrique Ribeiro CAMARGO

**Affiliations:** 1 PhD, Department of Bioscience and Oral Diagnosis, São José dos Campos Dental School, São Paulo State University (UNESP), São José dos Campos, SP, Brazil.; 2 PhD, Associate Professor, Department of Dental Materials and Prosthodontics, São José dos Campos Dental School, São Paulo State University (UNESP), São José dos Campos, SP, Brazil.; 3 PhD, Department of Physiotherapy, Superior School of Cruzeiro, Cruzeiro, SP, Brazil.; 4 PhD, Full Professor, Department of Bioscience and Oral Diagnosis, São José dos Campos Dental School, São Paulo State University (UNESP), São José dos Campos, SP, Brazil.; 5 PhD, Department of Restorative Dentistry, São José dos Campos Dental School, São Paulo State University (UNESP), ,São José dos Campos, SP, Brazil.

**Keywords:** Calcium hydroxide, Castor oil, Pulp capping, Rats

## Abstract

Castor oil bean cement (COB) is a new material that has been used as an endodontic sealer, and is a candidate material for direct pulp capping.

**Objective:**

The purpose of this study was to evaluate the biocompatibility of a new formulation of COB compared to calcium hydroxide cement (CH) and a control group without any material, in the subcutaneous tissue of rats.

**Material and methods:**

The materials were prepared, packed into polyethylene tubes, and implanted in the rat dorsal subcutaneous tissue. Animals were sacrificed at the 7th and 50th days after implantation. A quantitative analysis of inflammatory cells was performed and data were subjected to ANOVA and Tukey's tests at 5% significance level.

**Results:**

Comparing the mean number of inflammatory cells between the two experimental groups (COB and CH) and the control group, statistically significant difference (p=0.0001) was observed at 7 and 50 days. There were no significant differences (p=0.111) between tissue reaction to CH (382 inflammatory cells) and COB (330 inflammatory cells) after 7 days. After 50 days, significantly more inflammatory cells (p=0.02) were observed in the CH group (404 inflammatory cells) than in the COB group (177 inflammatory cells).

**Conclusions:**

These results demonstrate that the COB cement induces less inflammatory response within long periods.

## INTRODUCTION

One of the most important objectives of pulp preservation by direct pulp capping is the limitation of damage and to healthy function. Conservative endodontic techniques facilitate the maintenance of teeth with pulpal alterations, minimizing the unwanted sequelae of their unplanned extraction^13^.

In direct pulp capping procedures, a biocompatible or bio-inductive material is placed onto the exposed pulp tissue, preserving its vitality, stimulating the repair process, and promoting the formation of hard tissue barrier^9^. Biocompatibility is as important as the physical and chemical properties when selecting a material for endodontic therapy because of the direct contact with the vital tissue. However, some currently used pulp capping materials have a tissue-irritating potential^17^.

Calcium hydroxide (CH) cement presents properties such as low cytotoxicity, high pH^18,29^ and antibacterial action^10,25^. CH has been the material of choice for direct pulp capping because it seems to stimulate a rapid differentiation of odontoblast-like cells that form a hard tissue barrier in the pulp^10,25^. On the other hand, this action is not exclusive of CH and this materials suffers mechanical wear and solubility for long periods^9^.

The castor oil bean (COB) (*Ricinus communis)* is polyester formed by an amino radical, which confers bactericidal effect and has biocompatibility with living tissues^3^. It has great potential to facilitate tissue healing, excellent structural properties, low cost and does elicit toxic effects. COB^14^ has been tested in rabbits as a matrix for bone and joint replacement. After 40 days of surgery, the histological examination showed absence of late inflammatory reaction and no signs of systemic toxic effects.

Carvalho, et al.^6^ (1997) analyzed histometrically the alveolar bone healing around castor oil bean (Poliquil; Polímeros Químicos, Araraquara, SP, Brazil) implanted immediately after tooth extraction. The material was biologically compatible, as it was progressively integrated into alveolar bone in the healing process.

Barros, et al.^1^ (2003) investigated *in vivo* the biocompatibility of *Ricinus communis* polyurethane with three different chemical compositions. Modification of the polymer’s chemical composition by the addition of calcium carbonate or calcium phosphate promoted matrix mineralization, these materials being more biocompatible than pure resin. Mastrantonio and Ramalho^22^ (2003) evaluated the subcutaneous tissue reaction in mice, after the implantation of castor oil bean with or without calcium carbonate and showed that both materials are biocompatible.

In endodontics, COB has been used in retrograde filling materials in paraendodontic surgeries, irrigating agents and endodontic sealers^27,28^.

The use of a certain material must be based on experimental and laboratory studies that prove its biocompatibility and other properties^15^. The development of newer biocompatible, bactericidal, inductive materials that promote tissue repair and present adequate sealing can result in longevity of pulp capping procedures^10^. In addition, material selection is important for the success or failure of these treatments^5,15^.

With this objective, some methods have been developed to evaluate the irritating potential of dental materials. The implantation of materials in the subcutaneous connective tissues of small experimental animals is considered an adequate methodology to determine the biocompatibility of endodontic materials^5,20,26^ , although it is known that some reactions observed in this test cannot be considered identical to those occurring in living dental tissues.

It is believed that the pulp reaction can vary with the use of different available products, depending on their biocompatibility, which could cause severe damage to this tissue^12^. For this reason, there is an interest to increase the knowledge of the biocompatibility of COB because this material can be a candidate for direct pulp capping. The purpose of this study was to evaluate the biocompatibility of a new formulation of COB compared to CH and a control group without any material, in the subcutaneous tissue of rats.

## MATERIAL AND METHODS

This study was performed in accordance to the ethical Principles of Animal experimentation (COBeA – Brazilian College of Animal experimentation) and was approved by the local Research ethics Committee (São José dos Campos Dental School) (process no. 003/2006-PA/CeP).

Forty-two male Wistar rats (*Rattus norvegicus*) aged 90 days and weighing 350 to 400 g were used. The animals were maintained with food and water *ad libitum*.

The tested materials were a COB-based cement (Poliquil; Polímeros Químicos, Araraquara, SP, Brazil) and a CH cement (Dycal; Dentsply Petrópolis, RJ, Brazil). The COB cement was prepared according to the manufacturers’ instructions, mixing liquid polyol (5 mL), liquid prepolymer (5 mL), and calcium carbonate (5 g) until homogenization was obtained. CH cement was hand-mixed according to the manufacturer’s directions.

Polyethylene tubes (10-mm long x 1.5 mm inner diameter) (Johnson & Johnson, São José dos Campos, SP, Brazil) were washed with 70% alcohol and distilled water, autoclaved, and filled with the experimental materials using a lentulo spiral (KG Sorensen, Barueri, SP, Brazil) at low speed. All carriers and glass plates used in the study were previously sterilized.

The sample comprised three experimental groups of 14 animals each, half of which were killed after 7 and half at 50 days. each animal received a polyethylene tube containing COB cement or CH cement in the dorsum. In the control group, the animals received an empty polyethylene tube in the dorsum. Figure 1 shows the schematic design of the methodology.

**Figure 1:** Schematic design of materials and methods

**Groups**	**Subgroups**	**Experimental materials**	**Period (days)**
COB (n=14)	A (n=7)	Castor oil bean cement	7 days
	B (n=7)	Castor oil bean cement	50 days
CH (n=14)	A (n=7)	Calcium hydroxide	7 days
	B (n=7)	Calcium hydroxide	50 days
Control (n=14)	A (n=7)	Empty tube	7 days
	B (n=7)	Empty tube	50 days

COB-castor oil bean; CH-calcium hydroxide.

For the surgical procedures, the animals were anesthetized by intramuscular administration of 38.5 mg/kg of ketamine HCL (Dopalen; Vetbrands do Brasil Ltda., Campinas, SP, Brazil), and 14.2 mg/kg of xylazine (Anasedan; Vetbrands do Brasil Ltda., Campinas, SP, Brazil). The back of the animal was shaved and cleaned with 1% iodine in ethanol. Incisions were made on the dorsum, and one subcutaneous pocket was carefully prepared by blunt dissection. The base of the pocket was located at 10 mm from the incision line. A tube containing cement was then placed into each pocket and the incision was closed with surgical gut sutures.

The animals were killed after periods of 7 and 50 days, and the tubes were removed along with the surrounding tissue and immersed in 10% buffered formalin.

After fixing for 48 h, the tissue was processed for paraffin embedding. The tubes were removed during this procedure. A paraffin block was oriented in such a way that it was parallel to the long axis of the tube, and serial sections were cut to a 5-µm thickness. The sections were stained with hematoxylin and eosin. Histological qualitative and quantitative analyses of the inflammatory response were performed on light microscope. Color images of stained sections were acquired with a high-resolution camera (Sony Cyber-Shot DSC-S85; Sony Com. Ind. Ltda., Tokyo, Japan) at 200x original magnification for histomorphometric analysis. Only 1 view (original magnification 200x) standardized total area analyzed. It was positioned in the exact center of end of tube in each one of the 5 semi-serial slides *per* rat.

A single investigator blinded to the groups examined all specimens. Inflammatory cells were counted with an automated image analysis software, a public domain image processing and analysis program (Image J, National Institutes of Health – US Department of Health and Human Services, Bethesda, Maryland, USA), using the point tool. The criteria of histological quantitative evaluation were based on microscopic aspects. Lymphocytes presented small, round, very darkly staining nuclei and little surrounding cytoplasm. Macrophages presented larger, paler, oval or bean shaped nuclei and a somewhat larger amount of cytoplasm. Neutrophils were easily identified because of their polymorphic shape nucleus. Other inflammatory cells were not obvious in this study. Immature fibroblasts displaying large oval nuclei and mature fibroblasts with fusiform nuclei were observed but were not quantified. A mean number of inflammatory cells were obtained for each animal. Data were subjected to descriptive and inferential analysis. The means of inflammatory cells were tested by two-way ANOVA. The control, CH cement and COB cement groups were compared considering the following factors as variables: material and period of sacrifice (7 or 50 days after implantation). When the ANOVA showed statistically significant difference, the Tukey’s multiple-comparison test was used. The level of significance was 5% for both tests.

## RESULTS

### Histological qualitative analysis

**Figure 2:**
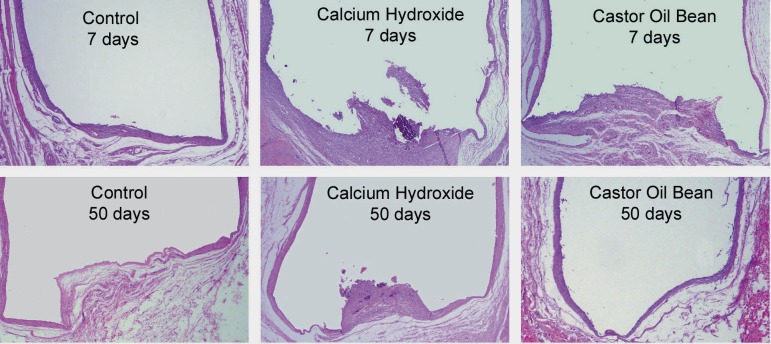
Panoramic pattern of each group (hematoxylin-eosin, original magnification 25x magnification), showing contact area of materials with the tissue through the ends of the open tube

The groups were compared qualitatively and no difference was observed between the CH and COB cement groups. Figure 2 shows an overview of the tube end with each material and experimental period.

### COB Cement Group

In the sections obtained at the 7th day, a moderate to severe chronic inflammatory process was observed, except for one specimen that presented mild intensity. Inflammatory infiltrate composed of mononuclear cells, mainly lymphocytes, was present near the material. In some specimens, there were also plasma cells, neutrophils and eosinophils in the inflammatory infiltrate. Only one case exhibited extravasation of COB granules, which were surrounded by numerous inflammatory cells. The connective tissue in contact with the material showed mild inflammatory reaction and a zone of necrosis was detected in few cases. The presence of multinuclear giant cells was not prominent in this experimental group. These cells appeared in a small number close to the material and presented three to five nuclei.

In the sections obtained at the 50th day, a mild to moderate inflammatory cell infiltrate was observed, mainly composed of lymphocytes and plasma cells. The presence of extravasated material in the subcutaneous tissue was associated with an increased inflammatory process. In most cases, the tissue was organized in a capsular arrangement with parallel collagens fibers, interspersed with fibroblasts and mature blood vessels. Multinuclear giant cells with three to five nuclei were found in most specimens, although they appeared in a small number and were distributed near the material.

### CH Cement Group

In the sections obtained at the 7th day, moderate to severe inflammatory cell infiltrate was observed. It was increased around the extravasated CH granules in the subcutaneous tissue. Except for one specimen that presented numerous polymorphonuclear leukocytes, mainly neutrophils, the tissue reactions showed an inflammatory infiltrate composed of lymphocytes and macrophages. Few plasma cells were observed. The macrophages were distributed around the overflow granules or diffusely in the granulation tissue. Sometimes, the cytoplasm of these macrophages presented some material particles. In some specimens, multinuclear giant cells with different amounts of nuclei and irregular cytoplasmic outline were found. The nuclei were distributed at random, characterizing a foreign body reaction. The connective tissue in contact with the material showed different degrees of reaction and a zone of necrosis was detected in many cases.

In sections obtained at the 50th day, moderate fibroblast and angioblastic proliferation was observed. The fibrous area presented a capsular arrangement with moderate amount of collagen fibers. Moderate to severe inflammatory infiltrate was verified, except in some specimens in which it was of mild intensity. The inflammatory cells were distributed around the extravasated CH granules. In this group, there was predominance of mononuclear cells, mainly lymphocytes and plasma cells, except for two cases exhibiting numerous neutrophils and eosinophils. Multinuclear giant cells and necrosis were not evident in this period.

### Control Group

Seven days after implantation, granulation tissue was observed in contact with the tube, presenting fibroblasts and new blood vessels. Moderate to mild inflammatory infiltrate composed of mononuclear cells, mainly lymphocytes and plasma cells, was observed. On day 50, a dense collagenous tissue was observed with scarce inflammatory cells, characterizing a fibrous capsule.

### Histomorphometric analysis

The analysis of the morphometric results shows that in the COB group, the number of inflammatory cells decreased from days 7 to 50.

Comparison of the mean number of inflammatory cells between the two experimental groups (CH and COB) and the control group by two-way ANOVA revealed significant difference (p=0.0001). There were no significant differences between tissue reaction to CH (382 ± 123.8 inflammatory cells) and COB (330 ± 106.9 inflammatory cells) after 7 days. However, after 50 days, the CH group presented a larger mean number of inflammatory cells than the COB group (404 ± 118.8 versus 176 ± 60.2 inflammatory cells). The mean number of inflammatory cells in the control group at 7 and 50 days was 69.0 ± 35.1 and 50.0 ± 33.5, respectively.

## DISCUSSION

Histomorphometric and/or quantitative analyses can be used to verify the inflammatory and repair phenomena and other reactions of dental materials in the subcutaneous tissue of rats. Some studies have used quantitative analysis^8,10,12,17,19^ because it is believed that, if made by calibrated examiners, it can determine reliable results. However, histomorphometric analysis based on counting of the number of inflammatory cells presents more reliable results than qualitative analysis with scores, for example^15,23^.

The Image J software is widely used in quantitative experiments^16^. There are many ways to apply computerized tools in morphometry. Semi automatic counting of inflammatory cells was used in this study to verify the intensity of the reaction caused by the implanted material. According to the obtained results, the simple qualitative analysis of specimens did not demonstrate any inflammatory differences between CH and COB implants. On day 50, the histomorphometric analysis showed that the COB cement presented a significantly smaller number of inflammatory cells than CH, and also showed no difference from the control group. On the other hand, qualitative analysis showed an inflammatory reaction similar to both cements, with mild to moderate intensity in the same evaluation period. Therefore, quantitative analysis was very important to show differences in tissue reaction to the tested cement.

CH cement produced minimum inflammatory reactions in a previous study that determined the flow characteristics and subcutaneous tissue reactions to CH and zinc oxide-eugenol endodontic sealers^15^. Fourteen days after implantation, the volume of tissue reaction was measured histomorphometrically. The highest flow values were obtained with CH cements, but the flow did not correlate with the degree of inflammatory response.

Kolokouris, et al.^19^ (1998) evaluated the *in vivo* biocompatibility of CH sealer in root canals. The intensity of reaction, initially severe, decreased on the 60th day, and this reduction continued progressively up to the 120th day.

In the present study, after 50 days, it was verified that the CH cement still showed an inflammatory process of moderate to severe intensity, mainly close to overflow CH granules. The presence of this material outside the tube increased not only the extension, but also the intensity of the inflammatory reaction. Therefore, before selecting a material for pulp capping procedures, it is important to know its mechanical properties. The ideal material should present low flow and should not be too much friable. Hydro C^®^ CH cement (Dentsply, Petrópolis, RJ, Brazil) exhibited the highest water sorption and solubility values after an evaluation of its mechanical properties^11^.

Although CH capacity of inducing the formation of a hard tissue bridge is an important property of pulp capping materials^18,29^ , in the present study the CH cement has less biocompatible than the COB cement. However, if we had used longer periods of evaluation, CH would be similar to COB cement. A large number of dental materials present cytotoxic effects when applied close or directly to the pulp, and the only material that seems to stimulate early pulp repair and dentin hard tissue barrier formation is CH. CH products are the best choice for conservative pulp treatments due to their therapeutic and biological potential, and the property of stimulating the formation of sclerotic and reparative dentin with a consequent protection of the pulp against thermal stimuli^24^.

Studies have persisted in the search for materials with high biocompatibility and good physicochemical properties, since the materials used in endodontic procedures can cause different reactions on pulp tissue^12^. The COB cement has been used in Medicine in the reconstruction, substitution or filling of bone defects presenting good results^14,21^.

In Dentistry, Calixto, et al.^3^ (2001) and Carvalho, et al.^6^ (1997) implanted a COB-derivative natural resin in the extraction wounds in rats to add information about the biocompatibility of this material. Their results were similar to those of the present study because after 6 weeks there was no persistence of inflammatory reaction, yet a small number of giant cells were observed in the tissue in contact with the material. Also, there was no foreign-body reaction or persistence of the inflammatory reaction in the study of Carvalho, et al.^7^ (1997). They evaluated histometrically the bone healing around polyurethane resin implants derived from castor bean and verified progressive osteogenesis in conjunction with a decrease in the fibrous capsule thickness.

Other authors have demonstrated that the incorporation of alkaline phosphatase to the *Ricinus communitis* polyurethane followed by synthetic body fluid incubation could be a useful alternative to improve the biological properties, as bone formation, of this polyurethane^2^.

According to Costa, Marcantonio and Hebling^8^ (1997), the COB cement presented acceptable biocompatibility under microscopic analysis when tested in subcutaneous implants in rats. The biocompatibility of COB cement has also described by Perassi, et al.^28^ (2004), who evaluated the tissue response of subcutaneous implants filled with COB cement and other endodontic sealers. The COB cement showed less tissue response than any other sealer in both experimental periods (7 and 50 days), which can be explained by the structure of this material with high pureness, lack of solvent, debris, stabilizers and degradation products present in other polymers, which would lead to adverse organic responses^28^.

Mastrantonio and Ramalho^22^ (2003) evaluated the subcutaneous tissue reaction in rats after the implantation of COB cement with or without calcium carbonate and showed that both materials induced mild inflammatory response after 7 days. Barros, et al.^1^ (2003) verified that the addition of calcium phosphate or calcium carbonate to the *Ricinus communis* polyurethane improved its biocompatibility implanted in rabbit femurs.

These results are according with those of the present study, which showed less inflammatory response and acceptable biocompatibility after 50 days of observation.

In summary, the findings of the present study indicate that the COB cement is a promising material. Furthermore, extracts of COB slightly induced cell proliferation and did not present genotoxicity without formation of micronuclei in V79 cells, or modification of the normal cell cycle in a previous *in vitro* study^4^ using primary human pulp-derived cells. However, new complementary studies are necessary to evaluating this material over the pulp tissue.

## CONCLUSION

These results demonstrate that the castor oil bean cement (COB) induces less inflammatory response within long periods.
